# Osseous oral hyaline ring granuloma mimicking a mandible tumor in a child with congenital agenesis of the corpus callosum

**DOI:** 10.4317/jced.53332

**Published:** 2017-02-01

**Authors:** Rodrigo Neves-Silva, Camilla-Borges Ferreira-Gomes, Natalia Palmier, Marcelo Brum-Corrêa, Oslei Paes-Almeida, Marcio Ajudarte-Lopes, Pablo Agustin-Vargas, Alan-Roger Santos-Silva

**Affiliations:** 1 DDS, PhD, Department of Oral Diagnosis, Oral Pathology and Oral Semiology Sections, Piracicaba Dental School, University of Campinas (UNICAMP); 2 MD, Department of Head and Neck Surgery, Oncology Center, Piracicaba

## Abstract

**Background:**

Hyaline ring granuloma (HRG) of the oral cavity is an uncommon disorder considered to be a foreign-body reaction resulting from implantation of food vegetable particles. Microscopically, it is characterized by the presence of structures of hyaline rings in an inflamed fibrous tissue background, which contains multinucleated giant cells.

**Material and Methods:**

We present the case of a 4-year-old boy diagnosed with a mandible osseous HRG, which showed clinical and tomographic aspects suggestive of an aggressive bone tumor.

**Results:**

The patient underwent surgical exploration and histopathologic analysis showed fragments composed predominantly of widespread dense connective tissue with an acute and chronic inflammatory infiltrate containing multinucleated giant cells and scattered areas of eosinophilic material associated with hyaline rings, strongly suggestive of vegetable particles. The eosinophilic material was positive for periodic acid-Schiff (PAS) and resistant to diastase digestion. These features led to diagnosis of osseous HRG. Scanning electron microscopy (SEM) analysis was performed for illustrative purposes and the multiple structures resembling vegetable particles were characterized in more detail.

**Conclusions:**

Although rare, this case highlights the importance of the clinician’s awareness regarding the existence of an osseous counterpart of HRG.

** Key words:**Agenesis of the corpus callosum, child, hyaline ring granuloma, intraosseous, mandible, pulse granuloma.

## Introduction

Hyaline ring granuloma (HRG) is a rare benign lesion of the oral cavity that affects almost exclusively soft tissues, with unclear and controversial etiology. The histopathological findings are characterized by the presence of a hyaline ring of eosinophilic amorphous material lying in a fibrous connective tissue with an inflammatory background and multinucleated giant cells (1,2).

Since 1971, HRG has been described in the literature under various terminologies, such as giant-cell hyaline angiopathy (1), hya-line ring granuloma (2,3), and pulse granuloma (4). The wide variety of terms proposed to describe this lesion reflects the fact that the nature of this granulomatous lesion is not yet fully understood.

Two opposing theories have been proposed in order to explain the nature of oral HRG (5). The exogenous theory believes that these structures are foreign bodies that might be leguminous food (pulse) or vegetable material (6), which were incidentally inserted into the socket following tooth extractions or through mucosal traumas, causing a granulomatous reaction (6). The second theory (endogenous) believes that localized acute vasculites takes on the characteristic of hyaline rings due to morphological changes in the walls of degenerated vessels (7). Nevertheless, this latter theory is unlikely since the recent literature contains increasing evidence to support legumes or other vegetable particles as the only cause of this particular lesion (3).

Oral HRG is mostly observed in adults, with the majority of cases affecting the soft tissue of edentulous areas (3). The clinical aspects may vary, but the lesions are generally characterized by recurrent oral swelling, which is tender on palpation, and covered by non-ulcerated mucosa. Moreover, patients may have discreet pain and purulent drainage (3).

Osseous cases of HRG have only rarely been reported in the English language literature, with a variable radiological appearance. Nevertheless, most of these cases are well-delimited radiolucent areas with only discreet bone erosion (3). The majority of the previously reported cases of osseous HRG are secondary to large bone lesions, particularly inflammatory periapical lesions and odontogenic cysts. Osseous HRG are exceptionally rare in the jaws of children, with only one previously reported case (1). Therefore, the aim here is to present an additional case of an uncommon osseous HRG that affected the mandible of an infant, and had imaginologic aspects similar to a destructive neoplastic bone lesion.

## Case Report

**Figure 1:**
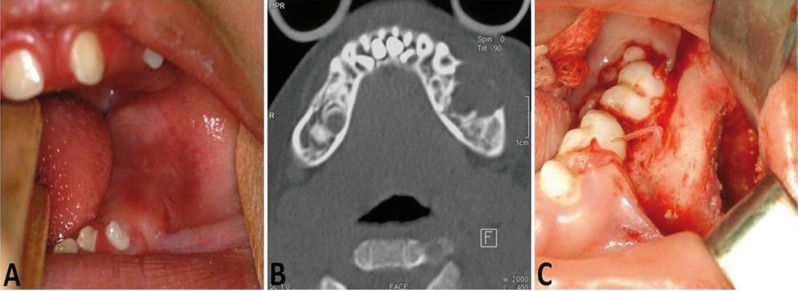
A) Intraoral view showing a buccal expansion affecting the left mandibular region. B) CT (axial section, bone window) displaying a hypodense image with bucco-lingual bony expansion and cortical bone thinning. C) Intraoperative image of the lesion showing impacted teeth and the erosion of the cortical bone.

A 4-year-old boy was referred for evaluation of a painless swelling in the mandible. According to his mother, 14 days before attending our clinic he presented high fever and edema on the left side of his face. His medical background included congenital agenesis of the corpus callosum causing vision impairment, cognitive low muscle tone and poor ability. He was taking phenobarbital (40 mg/day) to prevent recurrent seizure. No swelling was noted on his face on extraoral clinical examination. However, the patient presented lack of lip sealing. Intraorally, a fibroelastic swelling affecting the left body of the mandible measuring approximately 1.5 x 0.8 cm was noticed. The lesion was covered by normal mucosa without evidence of infection (Fig. 1A). In addition, the patient had gingival hyperplasia.

A computed tomography (CT) scan was performed and showed a hypodense osteolytic lesion on the left side of the mandible, extending from the distal surface of the deciduous canine to the molar regions (Fig. 1B). Furthermore, CT images (bone window) showed bucco-lingual expansion and cortical bone thinning on the buccal side, suggesting a cortical perforation, which indicated a locally aggressive lesion. Given the clinical and radiological aspects, the differential diagnoses included odontogenic tumors such as ameloblastoma unicystic or odontogenic keratocyst, a central giant cell granuloma and a malignant neoplasm, such as Langerhans cell histiocytosis or Burkitt´s lymphoma.

**Figure 2:**
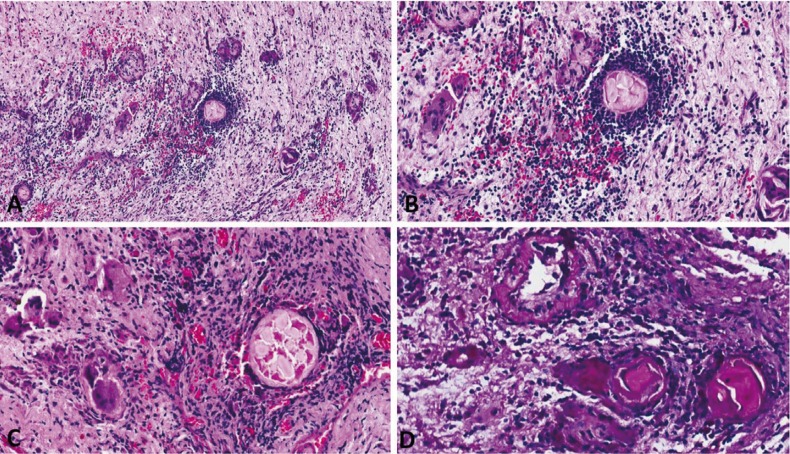
Microscopic aspects of HRG. A) A foreign body type granulomatous reaction with multinucleated giant cells arranged around hyaline-like eosinophilic areas in an inflamed fibrous connective tissue (original magnification; 20 x, H.E.). B,C) Hyaline ring structures surrounded by intense chronic inflammatory infiltrate and multinucleated giant cells (original magnification; 40 x, H.E.). D) Hyaline ring area positive for PAS and resistant to diastase digestion (40 x).

**Figure 3:**
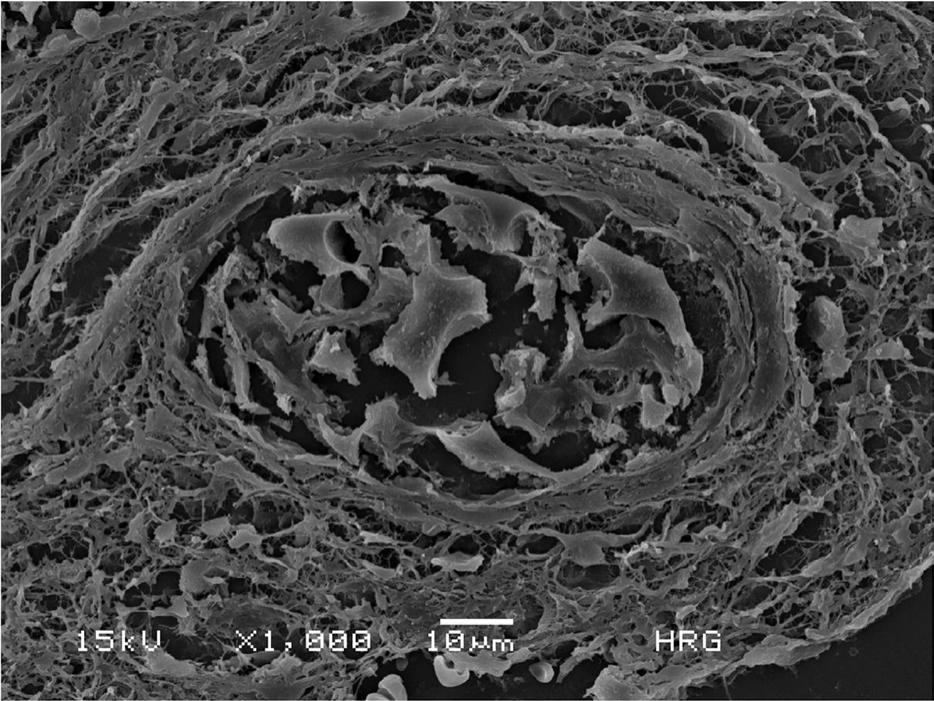
Scanning electron microscope SEM image of the HRG showing a round structure consistent with degenerated vegetable particles (1,000 x).

The patient underwent surgical exploration under general anesthesia and a yellowish fibrous material was completely removed (Fig. 1C). Histopathologic analysis showed fragments composed predominantly of widespread dense connective tissue with an acute and chronic inflammatory infiltrate containing multinucleated giant cells and scattered areas of eosinophilic material associated with hyaline rings (Fig. 2A). The hyaline rings formed central structures involving an amorphous material, strongly suggestive of vegetable particles (Figs. 2B,C). The eosinophilic material was positive for periodic acid-Schiff (PAS) and resistant to diastase digestion (Fig. 2D). These features led to diagnosis of osseous HRG. Scanning electron microscopy (SEM) analysis was performed for illustrative purposes and the multiple structures resembling vegetable particles were characterized in more detail (Fig. 3), supporting the histopathologic findings. The final diagnosis was an osseous HRG. After 6 months of clinical and radiological follow-up, no adverse effects or recurrences were observed. This study has been informed consent.

## Discussion

Despite the extensive discussion regarding the nomenclature and etiopathogenesis of HRG, it is most likely to be a consequence of implantation of leguminous seeds, resulting in a granulomatous reaction against this foreign body. According to the exogeneous theory, food debris may incidentally have been impounded on the mucosa in sockets following tooth extractions or secondary to bone lesions such as odontogenic cysts. These foreign materials then undergo attempted degradation by host phagocytic cells and produce eosinophilic and hyaline ring structures (6). Talacko and Radden (8) (1988) developed an experimental animal model of HRG by implanting food particles of leguminous origin into the orofacial region of rats and demonstrated that they were responsible for granulomatous reactions referred to as pulse granulomas and quite similar to the granuloma observed in the current report.

Whereas cases of HRG occur in the oral cavity (9), they have mainly been reported at other sites, such as the lungs and the gastrointestinal tract. The histopathologic features of oral HRG are similar to those lesions associated with vegetable particles found in intestinal fistulae, colonic diverticula and stomach ulcers, and those caused by aspiration in the lungs or lentil pneumonia in debilitated patients and children (10).

The most recent review of the English language literature regarding oral HRG found a total of 173 cases and the majority occurred in soft tissue (9). Clinical aspects are generally nonspecific painful swellings with inflammatory signs (3,4,9). Therefore, HRG is usually diagnosed microscopically (10). Oral osseous HRG are extremely rare, most being described in the walls of odontogenic cysts (11), or in periapical sites involving carious teeth or a history of failed endodontic therapy (9). No osseous case of HRG in an extraoral site has been reported in the literature to date.

Based on a review of the well-documented cases of oral osseous HRG, the majority of cases were diagnosed in adults (70%) and the most common location was the mandible of edentulous patients, and a large proportion of them occurred in association with odontogenic cysts (12).

Keirby and Soames (13) (1985) analyzed 24 cases of chronic periostitis and osteitis associated with hyaline bodies. Although all cases were histologically compatible with HRG, only 14 were genuinely osseous based on radiographic confirmation. However, we did not include these data in our review due to the lack of clinical information.

Unlike the reports found in the literature, the current case represents an unusual oral osseous HRG in a child, with atypical clinical and radiographic features that could lead to misdiagnosis and inadequate treatment, including an excessively aggressive approach. Due to a poor clinical history and limited clinical information, foreign bodies affecting infants may be confused with other lesions, including neoplastic processes (13).

Nuts and seeds are commonly reported as foreign bodies in children due to inhalation, with the risk of suffocation (14). On rare occasions they may induce an oral reactive lesion, mainly with intraosseous involvement. To our knowledge, this is only the se-cond case of osseous HRG in an infant reported in the English-language literature. The first case was a 6-year-old child with a radiolucent area beneath the resorbed roots of a deciduous molar that had previously received a steel crown; the authors discussed the possibility of a foreign substance and attributed the changes to acute vasculitis (1). A challenging aspect of the current case is how to explain how the legume particles could have become implanted so deeply in the jaw bone (mandible); the fact that a foreign-body reaction was able to achieve such large proportions is also intriguing. In this scenario, it is important to bear in mind that feeding and swallowing disorders are significant features in children with agenesis of the corpus callosum (15). Given these facts, we believe that one explanation for this extremely rare mandible osseous HRG would be previous and consecutive local trauma caused by the child or even secondary oral trauma caused by the caregivers, leading to deep implantation of legume particles and consequently an intraosseous foreign-body reaction.

In conclusion, the recognition of oral osseous HRG in children is important to enable a correct diagnosis and avoid unnecessary aggressive surgical treatment.
